# Evaluation of the knowledge and attitude of pharmacists about the national malaria control policy in southern Benin

**DOI:** 10.1186/s12936-017-1880-2

**Published:** 2017-05-31

**Authors:** Habib Ganfon, Giraud Ekanmian, Louis Amoussou, Emilie Daniel-Garcia, Aurel Constant Allabi

**Affiliations:** 1 Unité de formation et de recherche en Pharmacie, Faculté des Sciences de la Santé (FSS), Université d’Abomey-Calavi, 01 BP 188, Cotonou, Benin; 2 Centre d’Information Pharmaco-thérapeutique du Bénin, 06 BP 2610, Cotonou, Benin; 3 Réseau Médicament et Développement (ReMeD), 35 Rue Daviel, 75013 Paris, France

## Abstract

**Background:**

The national strategy against malaria in an endemic country should involve all the health stakeholders. In Benin, the private sector is rarely present in the activities of the National Malaria Control Programme (NMCP), and its surveillance system does not cover private sector outlets that are a non-negligible part of the healthcare system.

**Objective:**

The aim of this study was to describe the drug delivery practices within private pharmacies of Cotonou and Porto-Novo and the awareness of medicine providers concerning the national policy of malaria treatment.

**Methods:**

A survey was performed among pharmacy staff members responsible for dispensing medicines and providing advice to patients within pharmacies of Cotonou and Porto-Novo. Dispensing/pharmacy assistants (‘dispensators’) from 82 pharmacies in Cotonou and 19 in Porto-Novo were surveyed. Data entry was performed using Epidata 3.1 software and data analysis was carried out using SPSS software version 21.1. Chi square test was used to compare proportions. A significance threshold of 0.05 was defined for the p value.

**Results:**

46% of providers did not know the artemisinin-based combination therapy recommended by the NMCP for treating uncomplicated malaria. 58.7% were not able to recognize the gravity signs of malaria. 89.8% of dispensators were used to deliver an anti-malarial upon patient request, without prior biological confirmation as requested by the NMCP policy.

**Conclusions:**

Dispensing practices within the studied pharmacies from Cotonou and Porto-Novo were not in adequacy with the NMCP guidelines for uncomplicated malaria, which is a striking weakness in the training of drug providers on key elements of the guidelines for managing malaria. The NMCP needs to help dispensator from private pharmacies sector to standardize drug delivery practices according to its guidelines.

## Background

With more than 190 million cases in Africa, malaria is the most important tropical disease. In 2015, estimates suggested over 200 millions of malaria cases worldwide [1]. In 2013, the Benin country-profile, as well as the national health data repository, indicated an incidence rate of 17 per 100 inhabitants [2, 3]. In many countries, malaria has been estimated to represent up to 40% of the health costs, and 20–50% of hospital admissions [4]. Benin, like other malaria-endemic African countries, followed the recommendation of World Health Organization (WHO) and created a National Malaria Control Programme (NMCP) in 1982 [5]. Since then, the programme has been in charge of conception, organization, monitoring and evaluation of all activities deemed to control the disease. The creation of the NMCP has reduced the impact (mortality and morbidity as well as the economic impact) of malaria in Benin and other sub-Saharan countries over the last decade [6], with the help (financial and technical) provided by key partners, such as Global fund, United States Agency for International Development (USAID), WHO, Population Services International (PSI), Medical Care Development International (MCDI) [4, 5]. The development of resistance to major anti-malarial drugs (sulfadoxine–pyrimethamine, and chloroquine) has incited the country to adopt a new policy based on three keys interventions: the use of artemisinin-based combination therapy (ACT) for treating uncomplicated malaria; vector control with long lasting insecticide-treated nets (LLINs), domestic insecticide spraying, or mosquito larviciding; and chemoprophylaxis for pregnant women [5–7].

Notwithstanding, the evaluation of the system has revealed many weaknesses. A 2012 WHO report has shown that the surveillance system could detect only 10% of malaria cases because of incompleteness and inconsistency of reporting over time [8, 9]. Moreover, the activities of the key partners, including NMCP, are concentrated on public health facilities, whereas the private sector is not involved enough [10]. Indeed, the surveillance system set up by the NMCP in Benin collects data only from public and faith-based health facilities [11, 12]. Considering the supply of medicines to patients, public health facilities only account for 5% of anti-malarial providers [13]. In 2009, a study conducted in Benin by ACTwatch on behalf of PSI indicated that the presence of artemisinin-based combinations had increased in pharmacies (representing 52% of anti-malarial sales) [14]. Indeed, private pharmacies are healthcare providers responding to a request of an increasingly growing urban population, but their involvement in the actions and campaigns organized by the NMCP is almost inexistant [15]. Some reasons have been stated to justify the difficulties of a collaboration between private sector and the NMCP, including the fear of supplying the informal sector [14]. In a country where self-medication dominates medicine consumption [12, 16], the non-inclusion of private sector and especially pharmacies in the national strategy against malaria, may be greatly prejudicial to the effort makes toward the elimination of malaria.

The problem of adequacy and adherence between a national care policy and real practice of health stakeholders is not new. A study conducted in Nigeria by Ehrhun et al. in 2004 has shown the burden on the effort against malaria that can cause the unawareness of the national policy by the health stakeholders [17]. A similar study conducted in Bamako, Mali, in 2009 revealed that 77.4% of prescriptions were not in adequacy with the national treatment policy [18]. In the same period, a study by Robin in Benin [19] also showed inadequacies of care provider practices and national policies on malaria. This study has involved 14 private pharmacies, 10 public health centres, and 10 private health centres in Cotonou and Ouidah (Republic of Benin). The study indicated a lack of knowledge and unawareness of providers about the national protocol for the treatment of uncomplicated malaria [19]. Similar results were found in Ghana [20].

With the release of the WHO third guidelines for malaria cases management, it becomes interesting to study how healthcare workers complied with the previous recommendations in order to know how to implement the new one. The purpose of this study was to describe the drug delivery practices within private pharmacies of Cotonou and Porto-Novo, and if they comply with the NMCP new policy and recommendations.

## Methods

A descriptive cross-sectional study was conducted from January 2015 to October 2015.

### Study area

The study was conducted in urban regions, it included private pharmacies of Cotonou and Porto-Novo, and surroundings. In Benin there are overall 225 pharmacies with 98 in Cotonou and 24 in Porto-Novo. These regions account for 54% of all the pharmacies in the country.

### Study population

The study population consist of dispensator, it is the pharmacies staff members, those entrusted at the counter for dispensing medicines and counsel (‘dispensators’), pharmacist or auxiliaries. In each pharmacy, all the dispensators present at the counter at the time of the survey were accounted for. The study aim was explained to the agent and the oral consent was obtained before starting the survey.

### Data collection

The survey was conducted by two teams of students in pharmacy (5th year student) and pharmacists. The investigators responsibilities were to survey the dispensators (pharmacist or auxiliary) and accurately transcript their responses to the clients. The dispensator was only surveyed while standing at the counter during the investigators’ visit to the pharmacy. Whenever less than three dispensators were at the counter, the surveyor may ask for the other agents that were usually entrusted for delivery and counselling at the counter. When more than three dispensators were at the counter, all of them that accepted to participate in the study, were surveyed. Data collection material was a specific standardized questionnaire, pre-tested prior to the beginning of the study. Data collection aimed to be exhaustive targeting the 98 private pharmacies in Cotonou and the 24 in Porto-Novo.

### Data collected

- Status of the surveyed dispensator: licensed pharmacist, assistant pharmacist, auxiliary.
- Age, sex and number of year of experience as provider.
- For pharmacists (licensed or assistant): whether or not they have followed the course on the management of malaria cases at the pharmacy organized by the CIP.
- For auxiliaries: whether or not they have been trained by the pharmacist on the management of malaria cases at the pharmacy.
- Whether or not the NMCP guideline document was available in the pharmacy.
- Level of awareness on recommended association for treating uncomplicated malaria case.
- Whether or not they agree on the NMCP’ recommendations and, if not, the reason why.
- The dispensators attitude towards an uncomplicated case of malaria.
- Their knowledge of signs of severe malaria.
- The complains they receive from patient about anti-malarial drugs.
- Their attitude in case of reported adverse drugs effect.

### Statistical analysis

Data entry was performed by a team of two students in biostatistics and the accuracy and effectiveness of entry was controlled by a team of two pharmacists. Data entry had been performed using Epidata 3.1 software. Analysis was performed using SPSS (Statistical Package for the Social Sciences) version 21.1 Data was presented as proportion, mean, median, standard deviation, minimum and maximum. Chi square test was used to compare the variable accordingly. The probability of 5% will be used as the significance level.

## Results

### General characteristics of providers

A total of 101 pharmacies had participated in the study, (19 in Porto-novo, and 82) and 21 declined participation and a total of 285 providers have been surveyed which include 16 licensed pharmacists, 36 assistant pharmacists and 233 auxiliaries (82%). The sex ratio M/F was 0.3 in favour of women. The general characteristics of the surveyed providers are presented in Tables 1 and 2.

**Table 1:** General characteristic of the surveyed providers

Variable	n (%)
Quality of provider (n = 285)	
Licensed pharmacist	16 (5.6)
Pharmacist assistant	36 (12.6)
Auxiliary	233 (81.8)
Sex (n = 285)	
Females	216 (75.8)
Males	69 (24.2)
Years of experience (n = 285)	
≤2	66 (23.2)
2–5	115 (40.4)
5–10	58 (20.3)
>10	46 (16.1)

**Table 2:** General characteristic of the surveyed pharmacies

Variables	Mean	Standard deviation	Minimum	Maximum	Median
Ages of interviewed providers	30	8.3	18	65	28
Number of pharmacist (licensed and assistant) per pharmacy	2	1	1	6	2
Number of provider per pharmacy	8.8	4.8	1	38	8

### Awareness of providers regarding NMCP treatment policy

**Fig. 1:**
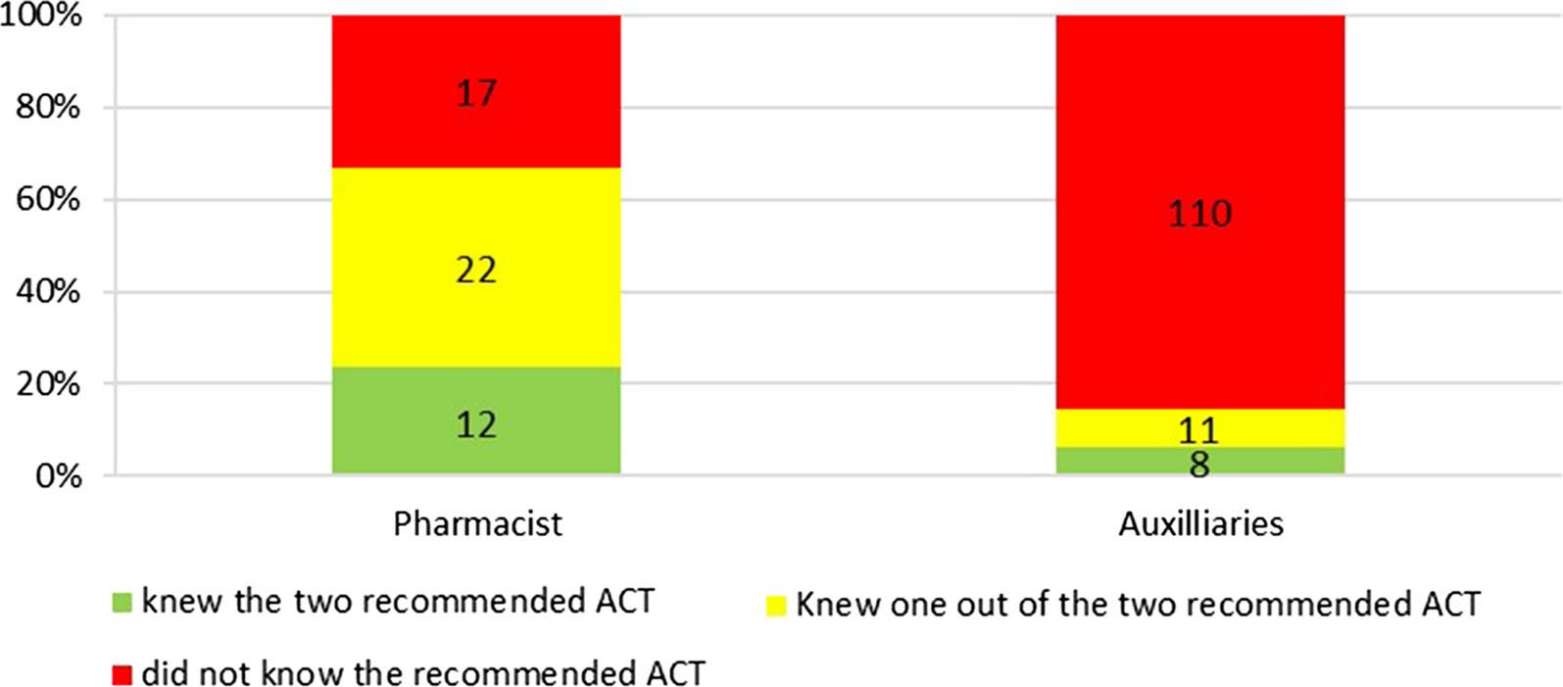
Proportion of providers according to their knowledge of the recommended ACT—(in *green*) providers that knew the two ACT recommended for treating uncomplicated malaria; (in *yellow*) providers that knew one of the two recommended ACT; and (in *red*) providers that did not know the recommended ACT

Less than half of drug providers 131/285 (46%) did not know the two forms of ACT recommended by the NMCP for treating uncomplicated malaria (artemether–lumefantrine and artesunate–amodiaquine). Only 7% (20/285) of providers were able to name precisely the two recommended ACT. Pharmacists have better knowledge of recommended ACT than auxiliaries; 23.1% (12/52) of pharmacist knew precisely the recommended ACT, while only 3.4% (8/233) of auxiliary knew it (Chi square = 12.724, p = 0.002) (Fig. 1).

**Fig. 2:**
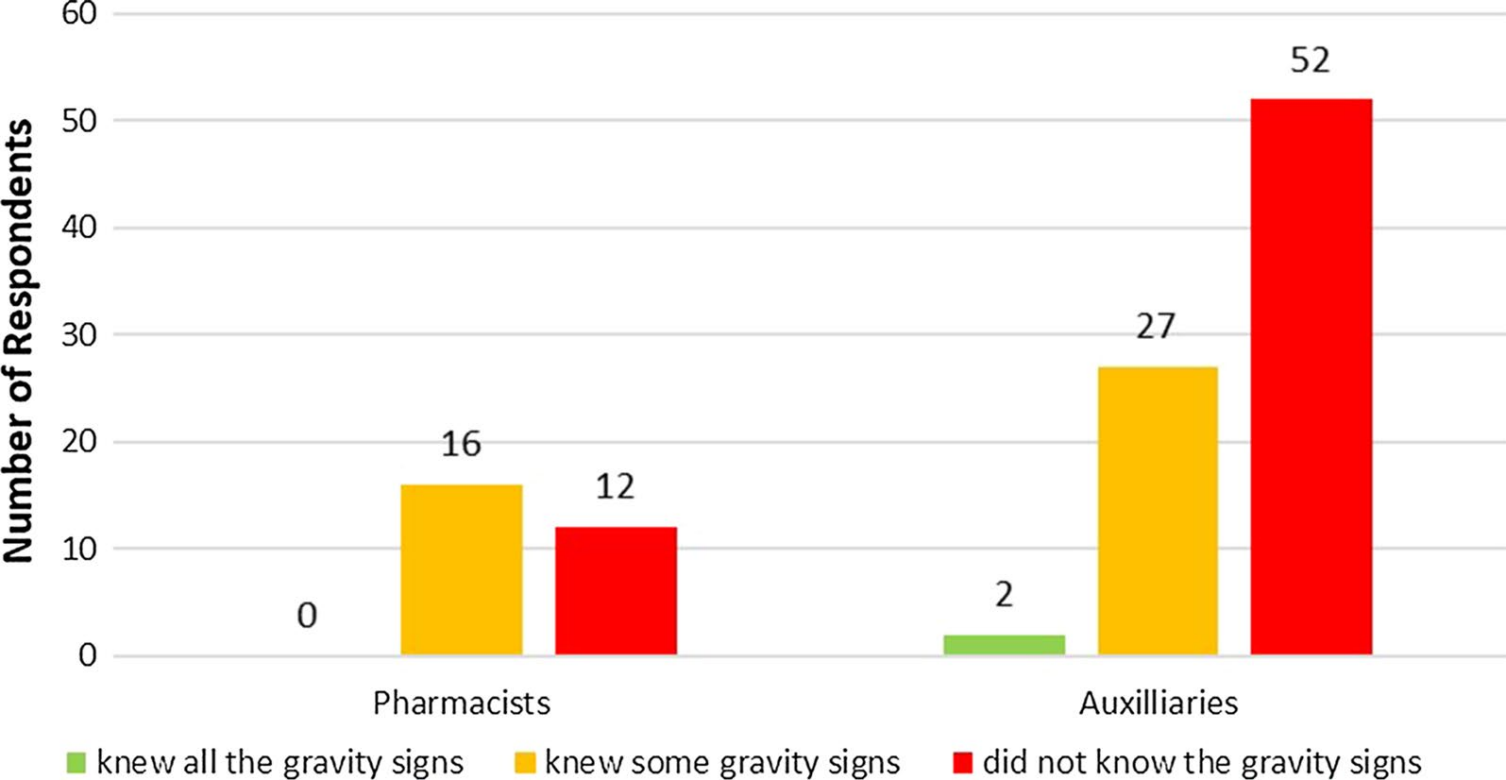
Proportion of providers according to whether they knew all the gravity signs (in *green*), knew some gravity signs (in *yellow*), or did not know the gravity signs (in *red*)

**Fig. 3:**
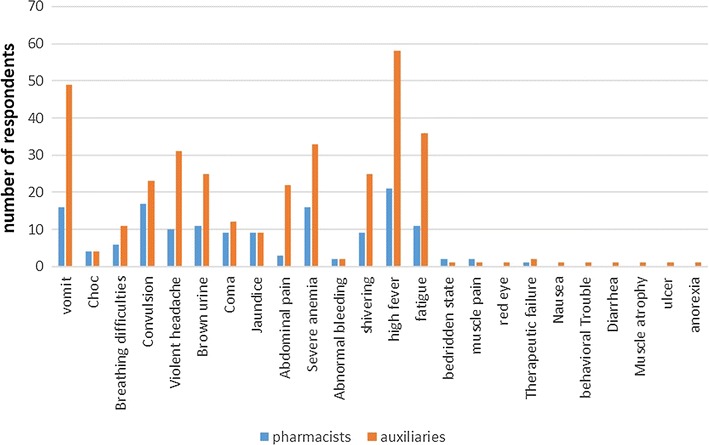
Gravity signs as enumerated by providers: (in *blue*) those enumerated by pharmacist and (in *brown*) those enumerated by assistants

About more than half of the providers (58.7%) (166/283) were not able to recognize the signs of severe malaria. Figure 2 shows to what extent providers knew about the gravity signs. Only 4/283 (1.4%) of the providers were able to enumerate all the signs for severe malaria. However, all pharmacists and auxiliaries failed to enumerate the gravity signs as defined in the NMCP document; even those able to recognize all the gravity sign have added some non-gravity signs as such. Figure 3 presents the gravity signs cited by the providers. High fever is the most cited gravity sign although it is not a sign of gravity.

NMCP document was present in the 66.7% (62/93) of the surveyed pharmacies, moreover 40.4% (31/52) of the pharmacist have followed the training on the monitoring of malaria case at pharmacy outlet organized by the NMCP 1 year before. 72.5% (169/285) of auxiliaries stated that they were trained on monitoring of malaria case at pharmacy outlet by the pharmacist (licensed or assistant), but the only (44/169) 26% of them showed the training notes.

89.8% (256/285) of the dispensators stated that they deliver anti-malarial drugs upon patient request. When they faced a suspicion of uncomplicated case of malaria, 6.3% (18/285) stated that they ensure that the patient performed and got a positive result to a biological test before the delivering of the requested anti-malarial drugs. And 3.86% (11/285) dispensator stated he referred the patient to a hospital. Only one pharmacy sold and performed RDT. About 10% of the pharmacies (11 of 101) sold RDTs.

When they faced a severe malaria case, 75.1% (214/285) of providers stated that would refer the patient to a hospital. However, 30.2% (86/285) of them stated they would deliver anti-malarial upon patient request. 1.4% (4/285) of providers would ask for a biological test before delivering anti-malarial drugs.

When asked their opinion about the NMCP guidelines for malaria treatment, the majority of respondent (94.5%) agreed with the document; of the 5.5% of providers that did not agree with the NMCP policy, one doubted about the efficacy of artemether–lumefantrine, two invoked the medicine cost, two mentioned tolerance problems, and one was concerned about abusive use of artemether–lumefantrine.

### Pharmacovigilance concern

**Fig. 4:**
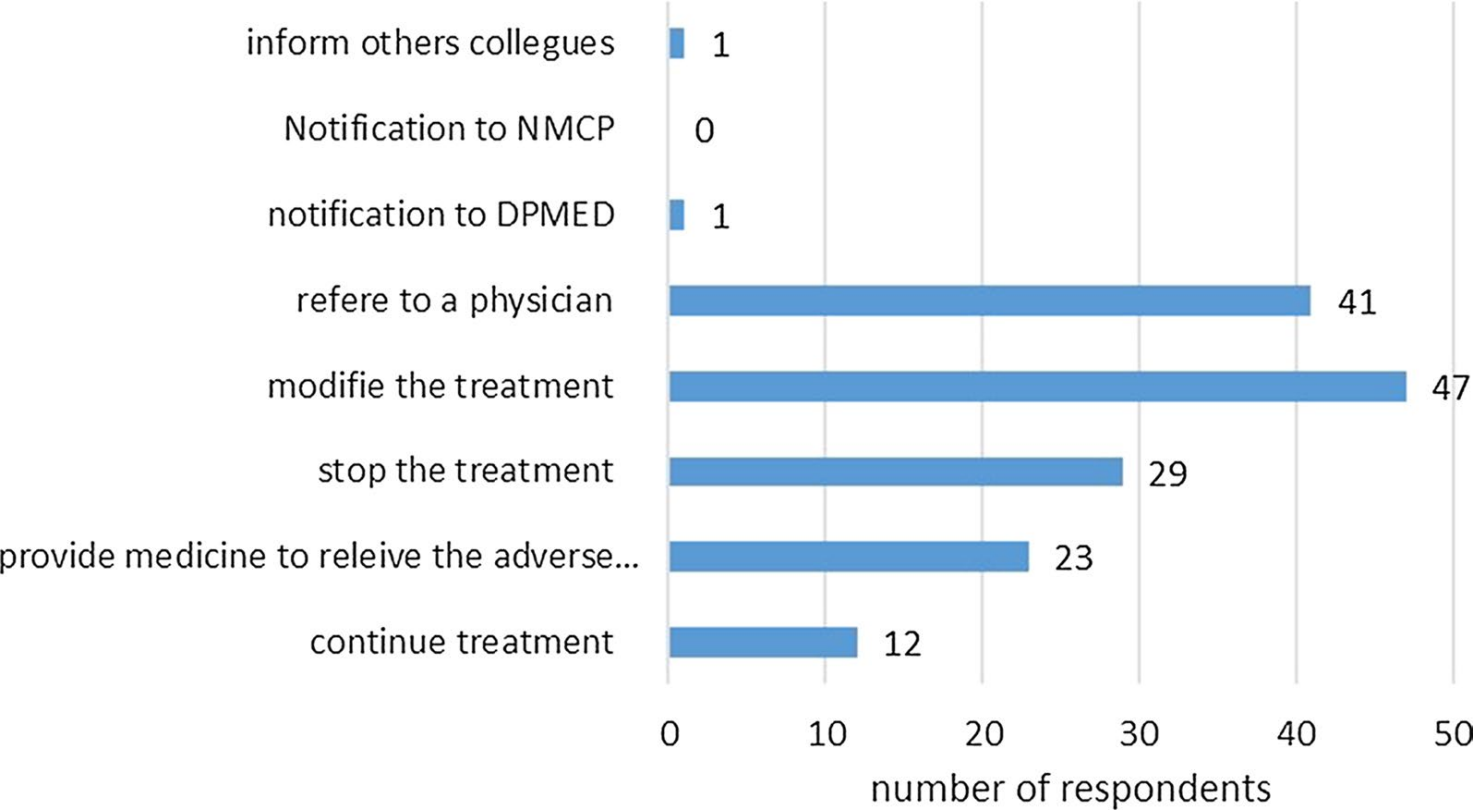
Chart presenting the different actions the prescribers are used to do whenever someone reports an adverse event

About half (47%) (134/285) of dispensators stated that patients complain about ACT. More than half (54%) of these complaints were about a feeling of inefficacy of ACT; adverse effects account for 43 and 3% of the complaints were about inadequacy of the galenic form. None of the dispensators stated they notified reported adverse events to the regulatory authority, or pharmaceuticals laboratory. Figure 4 summarizes the action taken by the dispensator in case of reported adverse event.

## Discussion

A first striking point of the result is the few number of pharmacists present at the counter, only 18% of personnel in charge of delivering medication and providing counselling are qualified pharmacists. The problem of compliance, as stated by the pharmaceutical policy, is not new, Robin and Gazengel had already described this in the study conducted in 2009 [19]. This absence was a root of numerous problems including the incentive for patient to prefer the informal sector.

The majority of dispensators, no matter their quality, are likely to provide anti-malarial drugs upon patient request regardless of the existence of a biological confirmation. Of course, this is at variance with the recommendation of the NMCP, particularly now with the launch of the 3T (track, test, treat,) initiative by WHO and Roll Back Malaria, that encourages the use of a standard malaria microscopy or the use of a quality-assured rapid diagnostic test (RDT) for universal confirmatory malaria diagnosis prior to treatment [21–23]. In numerous settings, the usage of a biological test before starting a malaria treatment has been found a cost effective strategy [24–26]: however these studies were conducted in hospital models, that do not consider patient’s self-medication. The trivialization of malaria in sub-Saharan countries incites population to go for self-medication to treat the disease; the availability and low price of monotherapy used before the introduction of ACT, as well as the previous protocol that recommended to treat any case of fever as malaria, contribute to this fact. The concern is the availability of RDT in pharmacies. The NMCP has implemented it for free in the public health facilities. There are no data in Benin regarding the willingness of patient to pay RDT in pharmacy before starting anti-malarial treatment. In the settings where this study was conducted, data revealed an inconsistent adherence to negative test. It is urgent to measure the willingness to pay for a test.

Majority of providers did not know the recommended associations for first and second line of anti-malarial treatment; moreover, the majority cannot recognize signs of severe malaria (58.7%). The training provided to pharmacists might not be incriminated in this situation since they were able to diagnose and counsel safe and effective medicine to their patient. The real problem is compliance to the NMCP guideline; even though the problem of awareness in general might be evoked, related to a weak awareness on the concept of evidence-based health, or more precisely the concept of awareness of the existence of such guidelines, the study revealed the presence of the guidelines in the majority of the pharmacies. Therefore, the discussion should concern the willingness of pharmacists to keep their knowledge updated, and quit a routine practice. Yet this argument is valid for pharmacists, who in this study were the minority of providers, and their knowledge of the policy is relatively better than that of auxiliaries. It is important to mention that the majority of pharmacists had been given the NMCP guidelines document after a training on management of malaria by the CIP.

More than 50% of patient’ complaints about ACT are about the inefficacy of AL. The resistance to AL is a growing debate [27, 28]; however this result should be considered with caution, knowing the absence of any biology test prior to anti-malarial use. This implies a possible use of anti-malarials outside the correct indication (i.e. in the absence of malaria), and in this case it may well be ineffective.

## Conclusions

These dispensing practices observed in pharmacies That are not in adequacy with respect to the guidelines for management of uncomplicated malaria are mainly linked to a weakness in the training, and in the awareness of providers on the national guideline for malaria management, implemented by the NMCP. One of the key elements of the strategy developed by the NMCP, is based on the provision of quality ACT and their rational use; best guarantee of their preservation against the parasitic resistance. Non-rational use of ACT through private sector with non-aligned dispensing practices with the formal guidelines, weakened any conservation strategy implemented in the public. This is why the NMCP should work intensively to standardize practices in both public and private sector because there is no private malaria apart from a public one.

## Abbreviations

ACT: artemisinin-based combination therapy
AL: artemether–lumefantrine
NMCP: National Malaria Control Programme
RDT: rapid diagnostic test
WHO: World Health Organization
